# Giant Mature Primary Retroperitoneal Teratoma in a Young Adult: Report of a Rare Case and Literature Review

**DOI:** 10.1155/2014/930538

**Published:** 2014-11-19

**Authors:** Walid Sasi, Giuseppe A. Ricchetti, Laila Parvanta, Robert Carpenter

**Affiliations:** ^1^St George's, University of London, Cranmer Terrace, London SW17 0RE, UK; ^2^St Bartholomew's Hospital, West Smithfield, London EC1A 7BE, UK; ^3^Homerton University Hospital, Homerton Row, London E9 6SR, UK; ^4^University College Hospital, 235 Euston Road, London NW1 2BU, UK

## Abstract

Teratomas are neoplasms of the embryonic tissues that typically arise in the gonadal and sacrococcygeal regions of adults and children. Primary adult retroperitoneal teratomas are rare and demand challenging management options. We report a case of a unilateral primary retroperitoneal mature cystic teratoma mimicking an adrenal mass in a 28-year-old female patient. Complete resection of the mass was performed by a laparotomy approach. Because of the risk of malignancy, follow-up radiographic studies were performed to ensure the oncologic efficacy of resection. The patient remains free of recurrence to date.

## 1. Introduction

Primary mature teratomas are uncommon nonseminomatous germ cell tumors and are made up of well-differentiated parenchymal tissues composed of somatic cell types that are derived from two or more germ layers (ectoderm, mesoderm, or endoderm) [1–3]. They usually occur in midline (paraxial) structures. The most common sites are gonads (testes and ovaries) followed by extragonadal sites such as intracranial, cervical, mediastinal, retroperitoneal, and sacrococcygeal regions [4–6]. Retroperitoneal teratomas are rare and account for only 4% of all primary teratomas [4, 7, 8]. They are believed to arise as metastasis from the gonadal tissue rather than to represent true primary tumours [2] and are more common in childhood and rarely occur in adults [9]. Only a very few case reports have been documented in literature so far [10]. The majority of cases are asymptomatic, present with nonspecific complaints, or are identified incidentally on routine investigations. Surgical excision of mature (benign) teratoma is required for a definitive diagnosis by histopathological examination and is the mainstay of treatment [11]. Prognosis is excellent after complete surgical excision with an overall five-year survival rate of nearly 100% [12]. Here we report a case of unilateral primary mature cystic teratoma of the retroperitoneum mimicking an adrenal mass in an adult female patient.

## 2. Case Report

**Figure 1:**
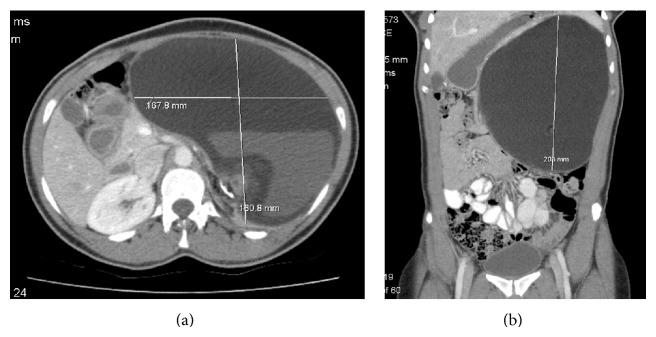
CT images of a giant left retroperitoneal teratoma: (a) cross-sectional view and (b) coronal view.

**Figure 2:**
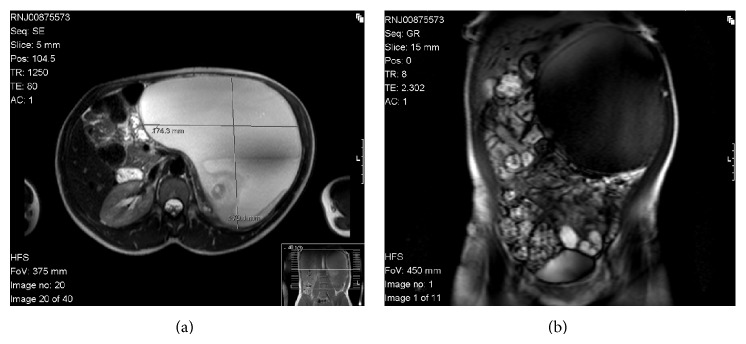
MRI images of the giant left retroperitoneal teratoma: (a) cross-sectional view and (b) coronal view.

A 28-year-old previously healthy female patient has attended our surgical clinic complaining of a chronic dull aching left-sided abdominal pain and mild abdominal distension for 1 year. During the course of investigations, she had an abdominal and pelvic CT scan and was found to have a 16.8 × 16 × 20 cm solid and cystic mass in the left retroperitoneum in the suprarenal area containing bone and multiple soft tissue densities (Figure 1). The tumour had calcifications, was not well enhanced, and was without any evidence of distant metastasis. Further assessment was performed using abdominal magnetic resonance imaging (MRI) (Figure 2). Preoperatively, we diagnosed the tumour as having originated in the left adrenal gland, because the normal adrenal gland could not be recognised by CT or MRI. Tumour markers, such as serum alpha-fetoprotein (AFP), carcinoembryonic antigen (CEA), lactate dehydrogenase (LDH), carcinoma antigen 125 (CA 125), neuron-specific enolase (NSE), and carbohydrate antigen 19-9 (CA 19-9), were examined because we also thought it might have been possible that the tumour did not originate from the adrenal gland.

**Figure 3:**
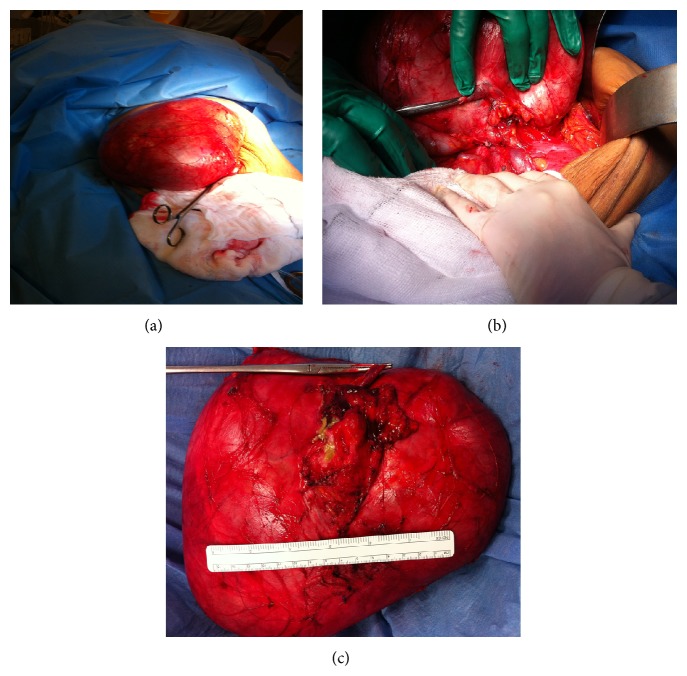
The giant teratoma at laparotomy ((a) and (b)) and after excision (c).

The patient underwent resection of the lesion through a midline laparotomy approach. The retroperitoneal dissection was tedious and difficult, but the mass was excised in its entirety (Figure 3). The macroscopic examination revealed a well-circumscribed mass measuring 230 mm × 200 mm × 120 mm, with intact surface. The specimen weighed in excess of 2 kg. The capsule was intact and the residual adrenal gland was noted on the external surface measuring 60 × 45 mm and was distinct from the mass. On slicing the specimen, the mass was completely filled with sebaceous-like material with identified hair and protuberances. On slicing, bony areas were noted.

Microscopically, the mass was composed of a mixture of mature components including bone, squamous epithelium, glandular epithelium, and stroma including muscle with no identified undifferentiated or primitive neuroectodermal tumour- (PNET-) like elements.

Adjacent to the tumour was a normal adrenal gland, which appeared to be distinct from the tumour. Ovarian tissue was not seen. Adjacent paraganglial tissue and ganglioneuronal tissue were also noted. There were no malignant features and the tumour appeared completely excised.

The conclusion of the histopathological examination was that of a mature teratoma, which might have arisen in the retroperitoneum and become attached to the left adrenal gland.

Because these tumours usually represent metastasis from other primary sites, additional imaging with CT of the chest and breast and ovarian ultrasonography was performed. No other primary tumour was identified. Therefore, we diagnosed the mass as a primary retroperitoneal teratoma. The patient is currently under clinical and imaging follow-up, to identify any future possible recurrence.

## 3. Discussion

Only a few case reports of the retroperitoneal teratomas have been documented in the literature so far [10]. They are more frequently encountered at the left side, and the majority is asymptomatic, present with nonspecific complaints, or is identified incidentally on routine investigations [7, 8]. Retroperitoneal mature cystic teratomas are frequently characterized by a bimodal peak in incidence, occurring in the first 6 months of life and in early adulthood [2]. Primary retroperitoneal teratomas in adults are uncommon, with only 32 cases reported between 1937 and 1987 [2]. Of note, documented cases of adult retroperitoneal teratomas are often secondary sites of tumor genesis and mostly occur in males [13], particularly from a primary gonadal tumor [2, 14], but other primary tumour sites such as the breasts and lungs have also been recognised to metastasize to the retroperitoneum [2].

Primary retroperitoneal teratomas are very unusual neoplasms accounting for approximately 1–11% of all primary retroperitoneal neoplasms and typically occur in neonates, infants, and children age groups [9, 15, 16], and only 10–20% of these occur in adults more than 30 years of age [17]. Hence the primary retroperitoneal teratoma in our adult female patient is an extremely unusual presentation. Our patient had a retroperitoneal mature cystic teratoma that was not derived from any specific organ. True primary retroperitoneal teratomas in adults are usually found in the upper portion of the left kidney [2]. In the case of a retroperitoneal tumor, germ cell tumors should be considered and tumor markers examined before surgery [18]. The malignancy rate of 26% in adults is significantly higher than the 7% rate documented in children [2].

Furthermore, primary retroperitoneal teratomas involving adrenal glands are exceedingly uncommon accounting for only 4% of all primary teratomas [4, 7, 8] and can be mistaken for other histologically related lipomatous adrenal neoplasms. However, in any suspected retroperitoneal teratoma in close proximity to or involving the adrenal glands, an adrenal origin of the tumour should always be considered until proven otherwise.

During the evaluation of these tumours, it is important to have a high index of clinical suspicion combined with routine laboratory work-up and radiographic investigations.

Clinical presentations are variable and include nonspecific, abdominal/flank/back pain and obstructive gastrointestinal and genitourinary symptoms, as well as lower limb/genital swelling due to lymphatic obstruction. They can rarely present with complications such as secondary infections (abscess formation) [19], traumatic rupture leading to acute peritonitis [20], or malignant transformations [21]. Midline (paraxial) teratoma masses, with restricted mobility, can be easily detected on physical examination.

Retroperitoneal teratomas can express a diversity of serum tumor markers such as elevated AFP, CEA, and CA 19-9 [15, 22, 23]. These serum tumor markers are helpful in clinical practice and they can be used not only to monitor successful treatment, but also to detect relapse in patients with specific tumor marker-secreting teratomas.

Radiographic investigations play valuable roles in diagnosing teratomas. Plain radiographs (X-ray) can identify calcified elements in 62% of cases [15, 22, 23] whereas ultrasound (US) can greatly differentiate between cystic and solid elements. CT scans can better distinguish between fat (adipose tissue) and bone (calcified) masses [24]. By comparison, MRI scans can show better resolution of soft tissues, feasible identification of benign and malignant neoplastic features, and most importantly superior tumor staging assessment [25].

In addition to their diagnostic role, these imaging studies are also of utmost importance when it comes to planning the surgical treatment as they can display the precise location, morphology, and adjacent structures of the teratoma. This provides better preoperative planning and increased likelihood of complete removal of the tumour with less iatrogenic damage [11].

Surgical excision of benign (mature) teratoma is required for a definitive histological diagnosis and is the mainstay of treatment [11]. Prognosis is fortunately excellent after complete surgical excision with an overall five-year survival rate of nearly 100% [12]. Teratomas are largely resistant to radiotherapy and chemotherapy. Adjuvant radio- and chemotherapy are used only if malignant features of germ cell tumors are identified on histopathological examination [2].

Regardless of the benign histological nature of mature teratomas, close follow-up is recommended because the incidence of malignant transformation is approximately 3–6% [14]. In the present case, the patient was free of recurrence after 6 months of follow-up.

In conclusion, primary retroperitoneal teratoma is a rare entity in adults. Though being usually asymptomatic, large neoplasms can cause abdominal and flank pain. Preoperatively, the diagnosis can be established by its characteristic appearance by imaging modalities such as CT and MRI. However, although retroperitoneal teratomas can be radiologically recognised, it is important to note that masses in the suprarenal region are likely to be confused with adrenal masses, as in our case during the preoperative stage. The definitive primary treatment of retroperitoneal teratomas is surgical resection.
